# Patients with Encapsulating Peritoneal Sclerosis Have Increased Peritoneal Expression of Connective Tissue Growth Factor (CCN2), Transforming Growth Factor-β1, and Vascular Endothelial Growth Factor

**DOI:** 10.1371/journal.pone.0112050

**Published:** 2014-11-10

**Authors:** Alferso C. Abrahams, Sayed M. Habib, Amélie Dendooven, Bruce L. Riser, Jan Willem van der Veer, Raechel J. Toorop, Michiel G. H. Betjes, Marianne C. Verhaar, Christopher J. E. Watson, Tri Q. Nguyen, Walther H. Boer

**Affiliations:** 1 Department of Nephrology and Hypertension, University Medical Center Utrecht, Utrecht, The Netherlands; 2 Department of Internal Medicine, Division of Nephrology and Transplantation, Erasmus Medical Center, Rotterdam, The Netherlands; 3 Department of Pathology, University Medical Center Utrecht, Utrecht, The Netherlands; 4 Department of Physiology and Biophysics, Rosalind Franklin University of Medicine and Science, IL, United States of America; 5 Department of Vascular Surgery, University Medical Center Utrecht, Utrecht, The Netherlands; 6 Department of Surgery, Box 202, Addenbrooke's Hospital, and Cambridge NIHR Biomedical Research Centre, Cambridge, United Kingdom; Boston University Goldman School of Dental Medicine, United States of America

## Abstract

**Introduction:**

Encapsulating peritoneal sclerosis (EPS) is a devastating complication of peritoneal dialysis (PD). The pathogenesis is not exactly known and no preventive strategy or targeted medical therapy is available. CCN2 has both pro-fibrotic and pro-angiogenic actions and appears an attractive target. Therefore, we studied peritoneal expression of CCN2, as well as TGFβ1 and VEGF, in different stages of peritoneal fibrosis.

**Materials and methods:**

Sixteen PD patients were investigated and compared to 12 hemodialysis patients and four pre-emptively transplanted patients. Furthermore, expression was investigated in 12 EPS patients in comparison with 13 PD and 12 non-PD patients without EPS. Peritoneal tissue was taken during kidney transplantation procedure or during EPS surgery. In a subset of patients, CCN2 protein levels in peritoneal effluent and plasma were determined. Samples were examined by qPCR, histology, immunohistochemistry, and ELISA.

**Results:**

Peritoneal CCN2 expression was 5-fold higher in PD patients compared to pre-emptively transplanted patients (P<0.05), but did not differ from hemodialysis patients. Peritoneal expression of TGFβ1 and VEGF were not different between the three groups; neither was peritoneal thickness. Peritoneum of EPS patients exhibited increased expression of CCN2 (35-fold, P<0.001), TGFβ1 (24-fold, P<0.05), and VEGF (77-fold, P<0.001) compared to PD patients without EPS. In EPS patients, CCN2 protein was mainly localized in peritoneal endothelial cells and fibroblasts. CCN2 protein levels were significantly higher in peritoneal effluent of EPS patients compared to levels in dialysate of PD patients (12.0±4.5 vs. 0.91±0.92 ng/ml, P<0.01), while plasma CCN2 levels were not increased.

**Conclusions:**

Peritoneal expression of CCN2, TGFβ1, and VEGF are significantly increased in EPS patients. In early stages of peritoneal fibrosis, only CCN2 expression is slightly increased. Peritoneal CCN2 overexpression in EPS patients is a locally driven response. The potential of CCN2 as biomarker and target for CCN2-inhibiting agents to prevent or treat EPS warrants further study.

## Introduction

Chronic treatment with peritoneal dialysis (PD) results in morphological changes in the peritoneal membrane. These alterations include progressive fibrosis and neo-angiogenesis and are associated with increased solute transport and loss of ultrafiltration [1]–[10]. Encapsulating peritoneal sclerosis (EPS) could be considered as a severe, advanced form of peritoneal fibrosis for which a second hit is mandatory [11]–[13]. This condition, for which no preventive strategy or targeted medical therapy is available, is a significant threat to patients on PD with a reported mortality of 25 to 55% [14], [15].

The human pathogenesis of EPS is not fully understood, but animal studies strongly suggest that both transforming growth factor-β1 (TGFβ1) and vascular endothelial growth factor (VEGF) are key players in the early peritoneal membrane changes and are potential targets for intervention [16]–[23]. This was recently illustrated by Loureiro *et al*. [24], who demonstrated that intraperitoneal administration of TGF-β1-blocking peptides to mice exposed to PD fluid significantly decreased peritoneal fibrosis and angiogenesis and improved peritoneal ultrafiltration.

Although this study suggests that TGFβ1 inhibition could be a tool to prevent or treat EPS, direct and chronic inhibition of systemic TGFβ1 activity is probably not desirable in humans given the important role of TGFβ1 in immune regulation and tumor suppression [25]–[31]. In this respect, the main downstream pro-fibrotic mediator of TGFβ1 activity, connective tissue growth factor (CCN2), might be a more attractive target. This protein, which is also involved in neo-angiogenesis [32], is overexpressed in a variety of fibrotic disorders [33]–[35] and some studies also suggest a pathogenic role for CCN2 in the peritoneal membrane alterations caused by long term PD [36]–[38].

Peritoneal biopsy studies in humans, which investigate the expression of pro-fibrotic and pro-angiogenic factors including CCN2, are scarce. In the current study, we investigated the peritoneal gene expression of CCN2, TGFβ1, and VEGF in different stages of peritoneal fibrosis. We hypothesized that CCN2, TGFβ1, and VEGF are already upregulated in early stages of peritoneal fibrosis and further overexpressed in EPS. For this purpose, we compared peritoneal expression of these factors in PD patients to PD naive patients with end stage kidney disease (ESKD) and in EPS patients in comparison to PD patients without EPS.

## Materials and Methods

### Study design

We performed two cross-sectional studies between October 2008 and September 2012. In study 1, we investigated peritoneal CCN2, TGFβ1, and VEGF gene expression and peritoneal thickness in three groups of patients with ESKD. In study 2, we determined peritoneal mRNA levels for CCN2, TGFβ1, and VEGF in EPS patients in comparison to two groups of patients without EPS (PD patients and non-PD patients).

### Study populations

#### Study 1

We obtained biopsies of the parietal peritoneum during kidney transplant procedures performed in the University Medical Center Utrecht in 16 PD patients, in 12 hemodialysis (HD) patients, and in four patients who received a pre-emptive kidney transplant. In PD patients, peritonitis was excluded at the time of biopsy by effluent cell count of <0.1×10^9^/L and negative culture.

#### Study 2

We performed a study in collaboration with Addenbrooke's Hospital in Cambridge, UK, which is one of the two referral centers in the UK for EPS surgery. Peritoneal tissue was obtained in 12 consecutive EPS patients who underwent surgery to resolve severe bowel obstruction. These EPS patients were compared with 25 ESKD patients without EPS who underwent kidney transplantation in the University Medical Center Utrecht; 13 patients who were on PD (PD group) and 12 ESKD patients who were never treated with PD (non-PD patients). None of these patients had symptoms of intestinal obstruction or had macroscopic signs of EPS (evaluated during kidney transplantation).

All EPS patients fulfilled the criteria for EPS developed by the Ad Hoc Committee of the International Society for Peritoneal Dialysis [39] and had severe clinical symptoms related to intestinal obstruction in combination with radiological evidence. In all EPS patients, the diagnosis was confirmed macroscopically. None of the PD patients had peritonitis at the time of biopsy.

Demographic and clinical data were collected from all patients. Both studies were conducted in accordance with the Declaration of Helsinki and the Good Clinical Practice guidelines set by the International Conference on Harmonization. Study protocols were approved by the Ethics Committees of the University Medical Center Utrecht and of the Addenbrooke's Hospital. All study participants provided written informed consent.

### Sample collection and processing

Collection of peritoneal tissue during kidney transplantation was done using a standardized procedure. In brief, a suture loop was inserted through the outer layer of the parietal peritoneum. By using the suture to lift the peritoneum, an ellipse of 2 cm in length was excised and pinned onto a paraffin wax plate with the mesothelial surface uppermost. Afterwards, the peritoneal cavity was closed with a suture.

In EPS patients, peritoneal tissue was taken during a decortication procedure which was performed to release obstructed small bowel segments. This sample included the falciform ligament and adjacent parietal peritoneum since this is typically excised and discarded.

Immediately after sampling, half of the biopsy was snap frozen in liquid nitrogen for isolation of RNA. The other half was fixed with 4% formaldehyde. After fixation for 24 hours at room temperature, samples were routinely processed for light microscopy and embedded in paraffin.

For study 2, we collected plasma samples before the surgical procedure whenever possible. If ascites fluid was present in EPS patients during surgery, this was also collected for analysis. In PD patients, a standard four-hour peritoneal equilibration test was performed [40] with a 3.86% glucose solution just before transplantation when time allowed this. Plasma and peritoneal fluid samples were stored at −80°C.

Samples were examined by quantitative PCR, histology, immunohistochemistry, and sandwich enzyme-linked immunosorbent assay (ELISA).

### Quantitative PCR

Total RNA was extracted from frozen peritoneal tissue using RNeasy columns (Qiagen, Venlo, The Netherlands). After cDNA synthesis, determination of CCN2 (Hs00170014_m1), TGFβ1 (Hs00998133_m1), and VEGF (Hs00900055_m1) mRNA levels were assessed by quantitative real-time PCR using TaqMan Gene Expression Assays with commercially pre-designed probe and primers (Applied Biosystems, Foster City, CA, USA). TATA box binding protein (TBP) (Hs00427620_m1) and glyceraldehyde 3-phosphate dehydrogenase (GAPDH) (Hs02758991_g1) were used as internal reference in study 1 and 2, respectively.

### Histological analysis

Peritoneal thickness was determined by measuring the thickness of the submesothelial compact (SMC) zone on hematoxylin and eosin stained formalin-fixed four-µm-thick tissue sections. The SMC zone is the submesothelial interstitial layer between the basal border of surface mesothelial cells and upper border of peritoneal adipose tissue. All biopsies were digitalized using a microscope camera (Nikon Eclipse E800 microscope with DXM1200 digital camera, Tokyo, Japan) and analyzed by image analysis software (ImageJ 1.45, NIH, Bethesda, MY, USA). Peritoneal thickness was measured perpendicular to the serosal surface at ten randomly selected points by two independent, blinded examiners, and then the average peritoneal thickness (APT) was calculated as described by Honda *et al*. [9]. The mean of the two APT values obtained in this fashion was used for further analysis.

### CCN2 immunohistochemistry

To validate CCN2 expression at the protein level and to determine its exact localization, CCN2 immunohistochemistry was performed in EPS patients on four-µm-thick paraffin embedded sections and compared to non-EPS PD patients. These samples were treated with endogenous peroxidase block. Antigen retrieval was achieved by predigestion with Protease XXIV (Sigma, St. Louis, MO, USA). Sections were incubated with a goat-anti-CCN2 antibody (sc-14939, Santa Cruz Biotechnology, Heidelberg, Germany), followed by rabbit-anti-goat IgG (Dako, Glostrup, Denmark) and goat-anti-rabbit BrightVision-PO (Klinipath, Duiven, Netherlands). Bound antibody was visualized with NovaRed (Vector Laboratories, Burlingame, CA, USA).

### CCN2 ELISA

Plasma and peritoneal fluid samples were thawed on the day of use and any solids pelleted by centrifugation. Afterwards, the supernatant was diluted 1∶10 in assay buffer. CCN2 present in the plasma or peritoneal fluid was then quantified using an ELISA as previously reported [41]. Briefly, the ELISA plate was first coated with CCN2 L-20 antibody, as a specific trapping antibody (sc-14939, Santa Cruz Biotechnology Inc., Dallas, TX, USA). The plates were incubated with sample or CCN2 standard and afterwards washed before the addition of 20a anti-CCN2 antibody. After further washing, horseradish peroxidase-conjugated secondary antibody (goat anti-rabbit; Jackson ImmunoResearch, West Grove, PA, USA) was added, followed by horseradish peroxidase substrate (Enhanced K-Blue TMB substrate, 308175; Neogen Corp., Lexington, KY, USA). The color intensity was allowed to develop, and read at 650 nm using a microplate reader. All CCN2 levels are expressed as ng/ml.

### Statistical analysis

Data are presented as mean ± SD or median (interquartile range). Difference between groups were analysed by one way ANOVA or Kruskal-Wallis test where appropriate, with Bonferroni correction or Dunn's test for multiple post-hoc comparisons.

Spearman's rank correlation test was used to assess correlation coefficients.

For all comparisons, a P-value <0.05 was considered to be significant (two-tailed). Statistical analysis was performed using GraphPad Prism software version 5.02 for Windows.

## Results

### Patient characteristics

The clinical characteristics of the study population are summarized in table 1 and 2.

**Table 1:** Baseline characteristics of patients in study 1.

	Peritoneal dialysis patients (n = 16)	Hemodialysis patients (n = 12)	Pre-emptively transplanted patients (n = 4)	P-value
Age (years)	56 (42–62)	57 (47–63)	43 (35–48)	0.10
Gender (F/M)	9/7	2/10	2/2	0.10
Cause of kidney disease				
Glomerulonephritis	6 (37%)	5 (41%)	0 (0%)	
Renal vascular disease	1 (6%)	0 (0%)	0 (0%)	
Cystic kidney disease	4 (25%)	2 (17%)	3 (75%)	
Diabetic nephropathy	0 (0%)	0 (0%)	0 (0%)	
Congenital and hereditarykidney disease	1 (6%)	0 (0%)	0 (0%)	
Other	2 (13%)	2 (17%)	0 (0%)	
Unknown	2 (13%)	3 (25%)	1 (25%)	
Dialysis duration (months)	34 (12–55)	45 (6–60)	N/A	0.82
Residual renal function (yes)	6 (38%)	5 (42%)	4 (100%)	0.07
History of peritonitis (yes)	8 (50%)	N/A	N/A	
Ultrafiltration failure (yes)	0 (0%)	N/A	N/A	

Data are median (IQR) or number of patients (%)

Abbreviations: F, female; M, male; N/A, not applicable.

**Table 2:** Baseline characteristics of patients in study 2.

	EPS patients (n = 12)	Peritoneal dialysis patients (n = 13)	Non peritoneal dialysis patients (n = 12)	P-value
Age (years)	47 (40–54)	56 (52–62)	52 (45–62)	<0.05*
Gender (F/M)	4/8	7/6	3/9	0.31
Cause of kidney disease				
Glomerulonephritis	3 (25%)	3 (23%)	4 (33%)	
Renal vascular disease	1 (8%)	0 (0%)	0 (0%)	
Cystic kidney disease	1 (8%)	5 (38%)	4 (33%)	
Diabetic nephropathy	3 (25%)	0 (0%)	0 (0%)	
Congenital and hereditarykidney disease	2 (17%)	1 (8%)	0 (0%)	
Other	0 (0%)	1 (8%)	2 (17%)	
Unknown	2 (17%)	3 (23%)	2 (17%)	
PD duration (months)	67 (53–98)	26 (12–45)	N/A	<0.001
History of peritonitis (yes)	7 (58%)	6 (46%)	N/A	0.54
Ultrafiltration failure (yes)	4 (31%)	0 (0%)	N/A	<0.05
Renal replacement therapy when EPS				
Peritoneal dialysis	0 (0%)	N/A	N/A	
Hemodialysis	9 (75%)	N/A	N/A	
Kidney transplantation	3 (25%)	N/A	N/A	
				

Data are median (IQR) or number of patients (%).

* EPS vs. PD.

Abbreviations: F, female; M, male; PD, peritoneal dialysis; EPS, encapsulating peritoneal sclerosis; N/A, not applicable.

In study 1, none of the PD patients had a history of ultrafiltration failure (UFF) and HD patients were predominantly male. In study 2, EPS patients were younger (P<0.05) and had a longer cumulative duration of PD (P<0.001) compared to PD patients without EPS. At the time of diagnosis, none of the EPS patients were treated with PD, nine were on HD and three had a functioning kidney graft. Four EPS patients and none of the PD patients without EPS had a known history of UFF.

### Peritoneal CCN2, TGFβ1, and VEGF gene expression and peritoneal thickness in PD patients, HD patients, and pre-emptively transplanted patients (study 1)

**Figure 1:**
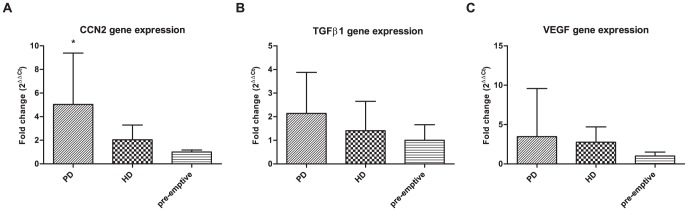
Peritoneal CCN2, TGFβ1, and VEGF gene expression in end stage kidney disease. Gene expression of CCN2 (**1A**), TGFβ1 (**1B**), and VEGF (**1C**) in peritoneum of peritoneal dialysis (PD), hemodialysis (HD), and pre-emptively transplanted patients. Mean±SD. * P<0.05 versus pre-emptive.

**Figure 2:**
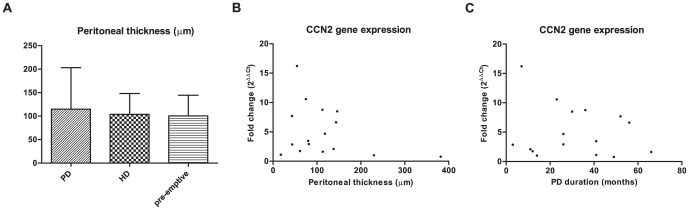
Peritoneal thickness, PD duration, and CCN2 expression in end stage kidney disease. Peritoneal thickness did not differ between peritoneal dialysis (PD), hemodialysis (HD), and pre-emptively transplanted patients (**2A**). Mean ±SD. In PD patients no correlation was found between CCN2 gene expression and peritoneal thickness (R = −0.24, P = 0.36) (**2B**) and between CCN2 gene expression and PD duration (R = −0.11, P = 0.67) (**2C**).

Peritoneal gene expression of CCN2 was 5-fold higher in PD patients compared to pre-emptively transplanted patients (P<0.05), but did not significantly differ from HD patients (figure 1A). Peritoneal gene expression of TGFβ1 and VEGF were not significantly different between the three groups (figure 1B and 1C). Peritoneal thickness also did not differ significantly between groups (figure 2A). In PD patients, no correlation was found between CCN2 gene expression and peritoneal thickness (figure 2B) or between CCN2 gene expression and PD duration (figure 2C). Also TGFβ1 and VEGF gene expression did not correlate with peritoneal thickness.

### Peritoneal CCN2, TGFβ1, and VEGF gene expression in EPS patients and PD and non-PD patients without EPS (study 2)

**Figure 3:**
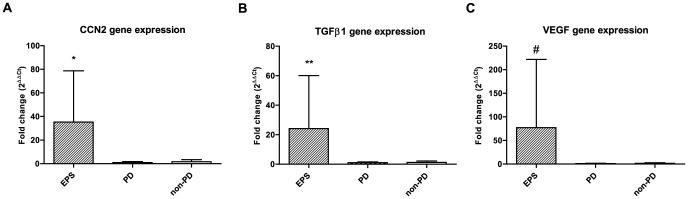
Peritoneal CCN2, TGFβ1, and VEGF gene expression in encapsulating peritoneal sclerosis. Gene expression of CCN2 (**3A**), TGFβ1 (**3B**), and VEGF (**3C**) in peritoneum of patients with encapsulating peritoneal sclerosis (EPS) compared to peritoneal dialysis (PD) and non-PD patients without EPS. Mean ±SD. * P<0.001 versus PD patients without EPS. ** P<0.05 versus PD patients without EPS. # P<0.001 versus PD patients without EPS.

Peritoneal gene expression of CCN2 was 35-fold higher in EPS patients compared to PD patients without EPS (P<0.001, figure 3A). CCN2 gene expression did not differ between the two groups of patients without EPS (figure 3A).

Peritoneal gene expression of TGFβ1 and VEGF were also higher in EPS patients compared to non-EPS PD patients (24-fold, P<0.05 and 77-fold, P<0.001, respectively), while this did not differ between the two groups of patients without EPS (figure 3B and 3C).

### Peritoneal CCN2 protein expression and CCN2 protein concentrations in ascites and plasma of EPS patients

**Figure 4:**
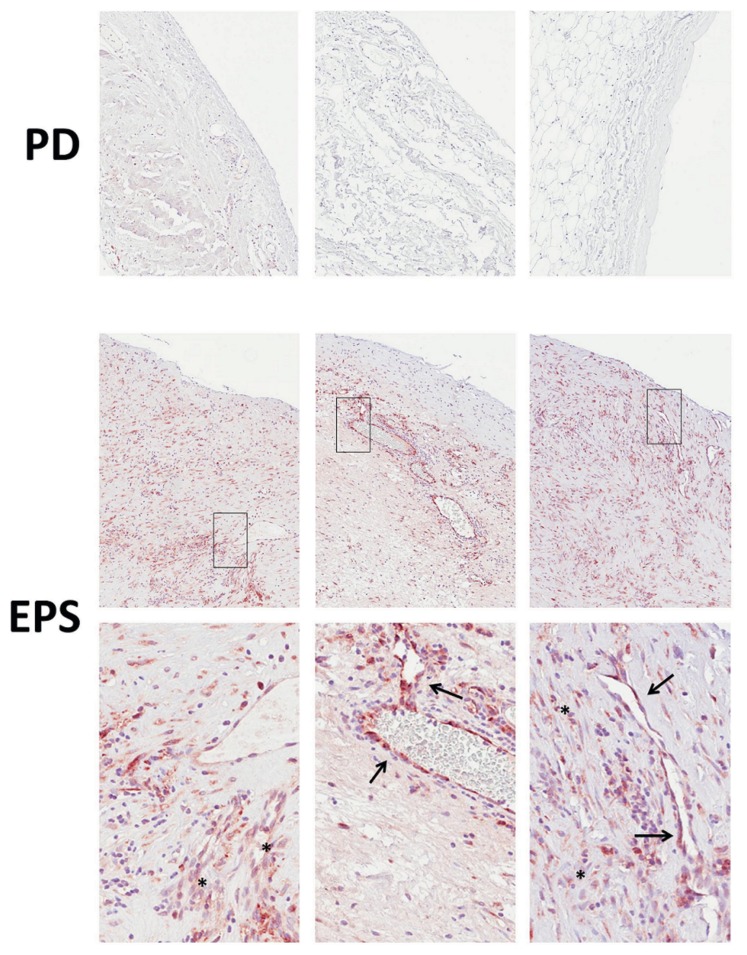
Peritoneal CCN2 protein expression in encapsulating peritoneal sclerosis. Immunohistochemistry for CCN2 in representative peritoneal biopsies of three peritoneal dialysis (PD) patients shows no significant staining (**upper panel**). In representative peritoneal biopsies of three patients with encapsulating peritoneal sclerosis (EPS), strong CCN2 staining is observed (**middle panel**). At higher magnification (**lower panel**), it is seen that CCN2 protein is mainly localized in endothelial cells (arrows) and interstitial fibroblasts (asterisks).

CCN2 immunohistochemistry could be performed on peritoneal samples of six EPS patients. In agreement with the increased CCN2 gene expression, we found a clear, prominent elevation in CCN2 staining in EPS patients compared to PD patients without EPS (n = 13) with localization mainly in endothelial cells and interstitial fibroblasts (figure 4).

**Figure 5:**
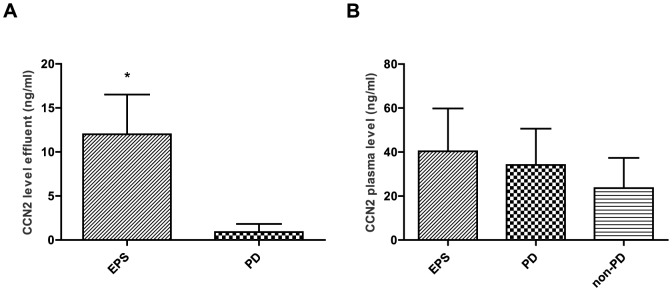
CCN2 protein levels in encapsulating peritoneal sclerosis. CCN2 protein levels in ascites of patients with encapsulating peritoneal sclerosis (EPS, n = 7) were higher compared to levels in dialysate of peritoneal dialysis (PD) patients without EPS obtained from a peritoneal equilibration test (n = 4) (**5A**). CCN2 protein levels in plasma of EPS patients (n = 6), in peritoneal dialysis (PD) patients (n = 13), and in PD naïve patients (non-PD, n = 12) did not differ between the groups (**5B**). Mean±SD. * P<0.01 versus PD.

Mean CCN2 protein concentration in ascites of EPS patients obtained in seven patients during surgery was 12.0±4.5 ng/ml. These levels were approximately 12-fold higher than levels measured in dialysate of four PD patients without EPS obtained from a standard four-hour peritoneal equilibration test (mean 0.91±0.92 ng/ml, P<0.01, figure 5A). In contrast, CCN2 protein levels determined in plasma did not differ between patient groups (40.4±19.5 ng/ml in EPS versus 34.1±16.5 ng/ml in PD without EPS versus 23.7±13.8 ng/ml in non-PD without EPS, P = 0.11, figure 5B). It should be noted that plasma samples were obtained in only six EPS patients.

## Discussion

Our study shows markedly increased gene expression of CCN2 (35-fold), TGFβ1 (24-fold), and VEGF (77-fold) in the peritoneal membrane of EPS patients compared to PD patients without EPS. In the latter group, we could at best demonstrate a very mildly (5-fold) increased peritoneal gene expression of CCN2 in patients with a median dialysis duration of 34 months compared to pre-emptively transplanted patients with ESKD, while TGFβ1 and VEGF gene expression were not increased. Peritoneal CCN2 protein expression, which was also markedly increased in EPS patients, was most prominent in endothelial cells and interstitial fibroblasts.

EPS is the most severe complication of treatment with PD and its pathogenesis is not exactly known. A two-hit theory has been proposed, with long term PD itself and the chronic use of bioincompatible PD solutions as first hits, leading to peritoneal fibrosis and possibly UFF. Severe peritonitis, discontinuation of PD or kidney transplantation have been proposed as second hits provoking fulminant EPS [13], [42].

Several *in vitro* and animal studies have focused on the pathogenesis of peritoneal fibrosis and sclerosis. These preclinical studies indicate that TGFβ1 and VEGF are key players in the development of both peritoneal fibrosis and EPS [16], [19], [21]–[24], [43] and that CCN2 expression in cultured human peritoneal mesothelial cells (HPMCs) and peritoneal fibroblasts could be induced by exposing these cells to PD solutions, glucose, glucose degradation products, and advanced glycation end products [36], [37]. However, biopsy studies in humans investigating the role of TGFβ1, VEGF, and CCN2 in peritoneal fibrosis and EPS in humans are scarce.

Using immunohistochemistry, Braun *et al*. studied the protein expression of TGFβ1, CCN2, and VEGF in the peritoneum of nine EPS patients who underwent surgery for bowel obstruction after a PD duration of 103±32 months [44]. For comparison, they studied patients on PD (n = 10, mean PD duration 19±28 months) and 10 non-renal surgical controls with normal peritoneum. Peritoneal tissue was obtained in the PD patients during elective surgery for hernias, leakage or catheter reinsertion. Compared to the surgical controls, peritoneal TGFβ1 was upregulated in patients on PD and in EPS patients, but the expression between the latter groups was not different. The peritoneal CCN2 expression was increased in PD patients compared to the surgical controls but did not differ from that in EPS patients. Interestingly, the peritoneal thickness in the PD patients without EPS was considerable (∼2000 µm) and comparable to that in EPS patients (∼2500 µm), indicating that the PD patients had a rather “sick” peritoneal membrane despite the relatively short PD duration. This may explain the lack of difference in TGFβ1 and CCN2 expression between PD patients and those suffering from EPS. Despite this similarity in peritoneal damage in the EPS and non-EPS groups in the study of Braun, the increase in VEGF expression was much more pronounced in the EPS group, pointing to an important role for this cytokine in the development of EPS. In agreement with this observation, the increase in VEGF mRNA expression was also more pronounced than that of TGFβ1 or CCN2 in the EPS patients in our study.

In PD patients with UFF, but without EPS, Mizutani *et al*. specifically investigated the peritoneal expression of CCN2 [38]. They studied the CCN2 mRNA levels in ESKD patients at the time of PD catheter implantation (uremic controls, n = 26), in incidental PD patients at the time of catheter removal for reasons other than UFF (n = 23) and in patients with UFF (n = 7). The peritoneal CCN2 mRNA expression in patients with UFF, who had been on PD for 9.4 years, was found to be increased 11.4-fold compared to the uremic controls, who also had a less thickened peritoneum than the UFF group (157 µm versus 308 µm). Interestingly, the CCN2 gene expression as well as the peritoneal thickness (155 µm) in the group of PD patients with normal ultrafiltration, who had been on PD for a mean of 3.7 years, were similar to that in the uremic control group, indicating that these patients had sustained little peritoneal damage despite PD treatment. This agrees with the very mild increase in CCN2 gene expression observed in our non-EPS PD patients.

The results of the above mentioned studies and our findings indicate that the fibrotic pathway involving TGFβ1 and CCN2 is markedly activated in the peritoneal tissues of patients with advanced peritoneal damage, resulting in either UFF (CCN2 in Mizutani's study) or EPS (both factors in the Braun *et al*. study and in ours). In addition, a very pronounced peritoneal upregulation of VEGF seems to be specific for EPS patients (Braun *et al*. and present study). In contrast, these growth factors are apparently not or only mildly (CCN2 in present study) upregulated in patients with a PD duration of 3 to 4 years who have a peritoneal thickness comparable to that of uremic and HD patients. It is of note that neither our study nor that of Mizutani *et al*. included a substantial number of patients with a PD duration intermediate between the 3 to 4 years of the control patients and the longer interval after which UFF or EPS develops. Therefore, it remains uncertain if activation of the fibrotic pathway involving overexpression of TGFβ1 and CCN2 occurs gradually or is a relatively abrupt and late phenomenon provoked by a second hit.

Our observation that both TGFβ1 and CCN2 are highly expressed in the peritoneum of EPS patients is in accordance with the current view that CCN2 is induced by TGFβ1 and acts as a cofactor to enhance the fibrotic actions of TGFβ1 [45]–[47]. The close interaction between CCN2 and TGFβ in the development of EPS was clearly demonstrated in an experiment by Wang *et al*. [46], in which only simultaneous intraperitoneal administration of TGFβ and CCN2 produced an EPS like phenotype in neonatal mice, whereas administration of either cytokine alone failed to produce any fibrosis. Although blocking of TGFβ1 seems a rational and effective approach to ameliorate peritoneal fibrosis [24], this may be unwarranted in view of the important favourable role that TGFβ1 plays in cell immunity, proliferation and differentiation [25]–[31]. Therefore, CCN2 may be a more appropriate target to prevent or reduce peritoneal fibrosis. Using small interfering RNA (siRNA) of CCN2, Liu *et al*. showed that the expression of CCN2 and VEGF induced by TGFβ1 can be inhibited in cultured HPMCs [48]. The same group showed that CCN2 knockdown using interference RNA also attenuated TGFβ1-induced extracellular matrix production and VEGF expression in HPMCs [49]. Phase 2 studies have indicated that EXC 001, an antisense oligonucleotide targeting CCN2, may be efficacious in reducing hypertrophic scar formation following surgery. Also, a humanized monoclonal antibody (FG-3019) directed at CCN2 has undergone safety trials in patients with diabetes mellitus and microalbuminuria [50], and is currently being tested in patients with idiopathic pulmonary fibrosis and liver fibrosis. Finally, it has recently been shown that CCN3, another CCN family member, works in a yin/yang manner to regulate CCN2 production and activity. In cell-based models of renal and skin disease, TGFβ1 and platelet-derived growth factor stimulated CCN2 activity and collagen deposition leading to progression of fibrosis. Exogenous CCN3 was able to block these effects [51], [52]. These findings with CCN3 have provided the basis for a third CCN2-targeted biological currently being developed as an anti-fibrosis therapy. Collectively, these data suggest that the application one or more of these biologicals, perhaps by the intraperitoneal route, may also be feasible in the PD patient to prevent or block progression of EPS.

Early detection of the development of EPS would be very important, especially in view of the potential preventive or therapeutic interventions. CCN2 is locally produced and present in dialysate [38], [53] and appears to correlate with the dialysate-to-plasma ratio of creatinine [38]. In addition, a positive relation was reported between the CCN2 concentration in PD fluid after an overnight dwell and PD duration in a cross-sectional study, albeit with a very low R^2^ value of 0.07. However, whether longitudinal follow-up of peritoneal CCN2 concentration or appearance rate has any value in predicting the development of peritoneal fibrosis or sclerosis remains to be studied. In view of the prominent overexpression of VEGF in EPS biopsies in our study and that of Braun *et al*. [44], it is disappointing that the longitudinally obtained dialysate VEGF concentration could not predict the development EPS in patients on PD [54], which makes it difficult to apply any preventive anti-VEGF therapy. Finally, TGFβ1 is also not a useful diagnostic marker for EPS as only an inactive soluble form is present in peritoneal effluent [55], [56]. To date, no study has measured CCN2 levels in ascites or in peritoneal effluent of EPS patients. We obtained ascites in 7 of the 12 EPS patients and found considerably higher CCN2 concentrations in ascites of EPS patients than in the effluent of non-EPS PD patients. In contrast, plasma concentrations of CCN2 did not differ. These high CCN2 levels in ascites in combination with peritoneal CCN2 overexpression agree with a locally driven response in EPS. However, we cannot rule out the effect of a difference in dwelling time on the local CCN2 levels in these groups. While the dwelling time in non-EPS patients was four hours, the “dwelling time” of ascites in our EPS patients was, for obvious reasons, unknown but undoubtedly longer than that.

In conclusion, peritoneal CCN2, TGFβ1, and VEGF expression are markedly increased in the peritoneal membrane of PD patients with EPS. Whether this increased expression is a late, sudden phenomenon induced by a second hit or a more gradual process correlated with PD duration remains to be determined. As CCN2 appears an attractive target for antifibrotic therapy, prospective, longitudinal studies are needed to determine whether the dialysate CCN2 concentration or appearance rate combined with peritoneal transport studies can predict the development of peritoneal fibrosis and sclerosis.
